# Optimizing Allelopathy Screening Bioassays by Using Nano Silver

**DOI:** 10.3390/life14060687

**Published:** 2024-05-27

**Authors:** Plamen Marinov-Serafimov, Irena Golubinova, Nadezhda Zapryanova, Ekaterina Valcheva, Bogdan Nikolov, Slaveya Petrova

**Affiliations:** 1 Agricultural Academy, Institute of Decorative and Medicinal Plants, Negovan, 1222 Sofia, Bulgaria; 2 Agricultural University, 12 Mendeleev Blvd., 4000 Plovdiv, Bulgaria; 3 University of Plovdiv Paisii Hilendarski, 24 Tsar Asen Str., 4000 Plovdiv, Bulgaria

**Keywords:** nanoparticles, nano solutions, inhibition, allelopathy, biotest

## Abstract

Nano solutions are widely used in medicine and also have the potential to be used when performing allelopathy screening studies. The present experiment aimed to test the effectiveness of colloidal nano silver Silver–Amber© with nanoparticles of 20 nm (>20 mg/L at a purity level of 99.99%) as a carrier of allelochemicals in laboratory conditions. The influence of eleven concentrations of Silver–Amber© (0.10, 0.20, 0.39, 0.78, 1.56, 3.13, 6.25, 12.5, 25.0, 50.0 and 100.0% *v*/*v*) on the germination and initial development of test plant *Lactuca sativa* L. in 0.75% agar medium was studied. Data revealed that when increasing the quantitative ratio of Silver–Amber©, an inhibitory effect on seed germination (from 37.8 to 94.3%) and on the plant growth (from 54.0 to 98.9%) appeared. Lower concentrations (0.63 to 0.04 ppm) had an indifferent to statistically unproven stimulatory effect on the germination and initial development of *L. sativa* (GI ranged from 88.7–94.6%). Therefore, nano silver can be used as carrier of allelochemicals in allelopathic studies in laboratory conditions.

## 1. Introduction

Conventional (inorganic) agriculture requires high inputs of raw materials and the use of innovative technological solutions to increase the efficiency and productivity of agricultural crops [1]. A recent review revealed that the economic losses in conventional agriculture are defined mainly as a consequence of the type and degree of weeding in cultivated areas [2]. Although widely used herbicides, on the one hand, have proven their efficacy, easy applicability and quick initial action to control the dominant weed species in cultivated areas, on the other hand, they have a high cost and are harmful to the environment. Many authors [3,4,5] have found that the intensive application of herbicides in crop lands enhances the compensatory processes in weed associations, and a calamitous increase of some invasive weed species has been observed as a result of their resistance to modern herbicides. Overuse of such chemicals leads to various negative consequences, i.e., antimicrobial resistance [6], food adulteration [7] and the decline of bees [8] and poses health risks for both animals and humans [9].

Hence, economic and environmental constraints in agricultural production require a search for alternative strategies and technological solutions to combat weeds. At the modern stage in herbological practice, a phytocenological approach based on biological (allelopathic) relationships has been implemented in weed management, aiming at ensuring the sustainable development of agrophytocoenoses with minimal environmental risk [10]. Allelopathic relationships in agroecosystems determine the potential to regulate weed rates through the synthesis and release of secondary metabolites (allelochemicals) from different plant parts [11,12]. Despite established dependencies in the dynamics of allelopathic relationships in plant communities, allelopathic interference is still underutilized in agricultural practice. The main reasons for this are the prevailing limitations in unifying experimental practices, mainly related to the use of different carriers and techniques of extraction of allelochemicals and their identification when performing experimental studies [13,14,15,16]. The obtained extracts from plant fresh and/or dry biomass are extremely unstable; thus, they create suitable conditions for the development of microorganisms that have a negative impact on the germination and initial development of test plants and there are problems in distinguishing the effects of allelochemicals from those of microorganisms. Therefore, it is necessary to store extracts at low temperatures [17,18,19] or as lyophilizates, which requires expensive laboratory equipment or the addition of antimicrobial compounds that are classified as chemical preservatives, which sometimes could also provoke an undesirable impact [20]. As such, there is a crucial need for new solutions that can address these problems with the storage of allelochemical extracts, as well as enhance their effectiveness.

In the last decade, nano solutions have been studied by a number of researchers in connection with their application in pharmacology, biotechnology, industry, agriculture, etc. [9,21]. Their large-scale application and usage in modern technological solutions is due to the unique physico-chemical properties of nanoparticles—small size, allowing them to penetrate deeply into different living tissues, large surface area to volume ratio, high reactivity, high carrier capacity and easy changes in their surface reactivity [9,22]. However, there is some evidence that the intensive use of nano-pesticides, nano-fertilizers, etc., poses a significant danger to soil quality [23]. Thus, the relationships between nano-materials and soil properties also still need to be studied. Many authors have demonstrated that silver nanoparticles are the most commonly used nanoparticles in nano solutions as they have a large surface area, very small size, high dispersion [24,25], low toxicity and proven antioxidant and antimicrobial properties [26,27,28]. Their exceptional antibacterial efficacy is a result of various physical and chemical interactions with bacterial cells—disruption of cell membranes, oxidative stress, enzymatic inhibition, ion imbalances and inhibition of bacterial growth and reproduction [9,29].

The scientific literature regarding the application of nano silver as a possible carrier of allelochemicals in allelopathic screening studies is scarce and the obtained experimental results are often contradictory [30,31,32]. Plant responses to nano silver exposure include reactive oxygen species production, increment of H_2_O_2_ levels in plant cells, activation of enzymatic defense systems, changes in pigment contents, etc. [32]. Such processes could result in abnormal morphology, metabolism disruption and many others, depending on the plant species and the nanoparticles’ properties—type (chemically synthesized, green synthesized or commercially produced), size, shape and concentration [28,31,32]. Wang et al. [33] found that all nano silver particles significantly decreased seed germination and seedling development, but those with larger sizes exerted stronger toxicity in both lettuce (*Lactuca sativa* L.) and jasmine rice (*Oryza sativa* L.), expressed as inhibition of leaf length and width. Another finding was that the effect of Canada goldenrod leaf extract was significantly enhanced in the presence of nano silver in the medium. Thus, as the first step, it is crucial to deeply understand the effect of silver nano-materials on the plant species used as test organisms in laboratory bioassays—both on seed germination and plant development—and if there is no negative influence, as the second step, we could assess their potential to be used as carriers of allelochemicals and/or antimicrobial agents.

Based on the abovementioned factors, the aim of the present study was to (i) analyze the effect of colloidal nano silver Silver–Amber© (nanoparticle size 20 nm, concentration >20 mg/L at purity level 99.99%) on the seed germination and initial development of *Lactuca sativa* L. test plants; and (ii) assess its possible application in allelopathic screening studies in laboratory conditions. Our research hypothesis was as follows: if we could find a silver nanoparticle concentration that did not impact the test plant’s germination and development, they could be applied as carriers of allelochemicals and thus enhance the performance of allelopathic bioassays.

## 2. Materials and Methods

The commercial product Silver–Amber©, Corby, UK (colloidal nano silver with nanoparticles <20 nm, concentration of >20 mg/L at a purity level of 99.99%) was used at the equivalent of 100% initial concentration and diluted with redistilled water to final concentrations of 0.10, 0.20, 0.39, 0.78, 1.56, 3.13, 6.25, 12.5, 25.0, 50.0 and 100.0% *v*/*v*.

*Lactuca sativa* L. cultivar ‘Great Lakes’ was used as a test plant due to its proven sensitivity to potentially toxic substances [34,35]. An adaptation of the ‘Rhizosphere Soil Method’ (RSM) of Fujii et al. [36] was applied in the experimental design.

**Figure 1:**
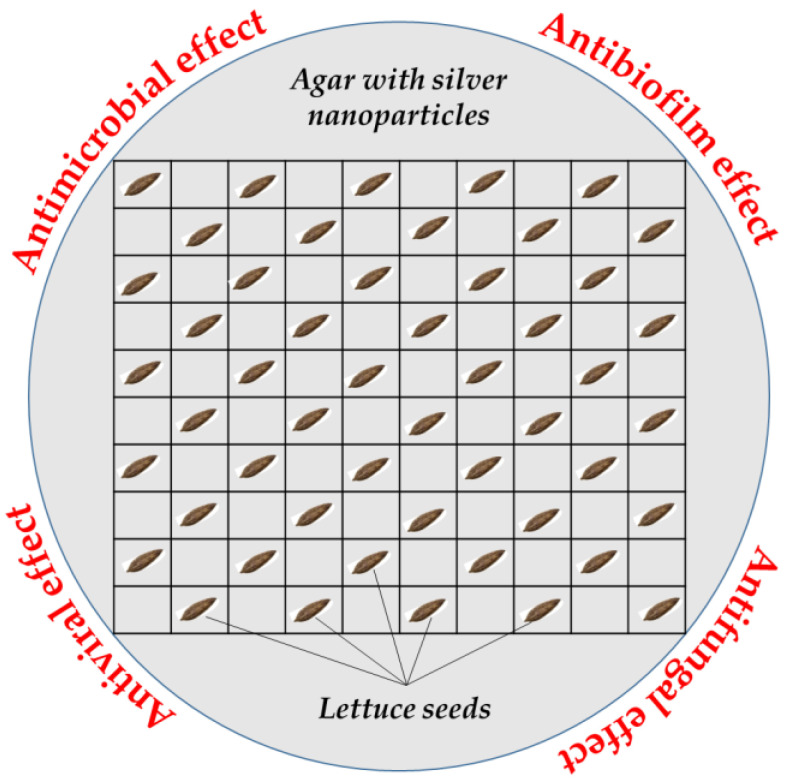
Schematic illustration for the preparation of experimental Petri dishes.

A semi-solid 0.75% agar was used as a growth medium for the test plant as follows: 0.45 g of agar was added to 60 mL of the tested concentrations of colloidal nano silver and tempered at 45 °C. Then, 20 mL of each of the thus prepared agars was pipetted into Petri dishes (90 mm). After the formation of the agar gel, 50 seeds of *Lactuca sativa* L. were placed in each petri dish and sealed with paraffin tape (Figure 1). Control variants were prepared in the same manner by using distilled water instead of nano silver solution. Each experimental variant was set in eight replicates. All experimental variants were incubated in a thermostat device in the dark at a temperature of 22 ± 2 °C for five days [37].

In order to prevent microbial contamination from the plant seeds, their surface was sterilized by a 0.2% *v*/*v* solution of sodium hypochlorite for 5 min, followed by washing four times with distilled water, then sterilized with 70% *v*/*v* ethanol for 2.5 min and washed four times with distilled water using a Buchner funnel [38].

Six different coefficients and indices have been applied, aiming at to reveal a complex analysis of the effect of colloidal nano silver on the germination and initial development of the test plant (*Lactuca sativa* L.), as shown in Table 1.

Raw data obtained were processed using the software products Statgraphics Plus for Windows Ver. 2.1 and Statistica Ver. 10 by one-way analysis of variance (ANOVA) at the level of statistical significance (*p* ≤ 0.001). Percent of germinated seeds (*GP_%_*) was transformed using arcsin (arcsin √x) [44].

## 3. Results

The colloidal nano silver from the commercial product Silver–Amber© exerted an inhibitory effect on seed germination of the test plant *Lactuca sativa* L. (lab bioassay) when compared to the control variant with redistilled water (Table 2). Raw data analyses revealed that the suppression was statistically significant (*p* ≤ 0.001) at the higher concentrations applied (from 1.56 to 100.0% *v*/*v*), while the lower concentrations (from 0.20 to 0.78% *v*/*v*) had a weaker to indifferent effect on the seed germination. An exception was found only at the lowest concentration—0.10% *v*/*v*, where a significant stimulating effect (*p* ≤ 0.001) was found. Furthermore, the applied concentrations of colloidal nano silver showed a non-monotonic concentration–inhibition curve (NMCIC) on the laboratory germination of *L. sativa* seeds. The percentage of inhibition varied from *I_%_* = −7.91% (at the lowest concentration, 0.10% *v*/*v* of colloidal nano silver) to *I_%_* = 26.33% (at the highest concentrations, 50.0 and 100.0% *v*/*v* of the nano silver solution). As the concentrations of nano silver increased from 0.20 to 1.56% *v*/*v*, the inhibition percentage increased from 1.5 to 3.1 times (*I_%_* = 7.13 ÷ 22.18%), while in the range from 12.5 to 25.0% *v*/*v*, a retention effect was found (*I_%_* from 17.77 to 22.18%) (Table 2).

Regression analysis was performed, aiming to discover the relationships between the seed germination and concentration of nano silver applied. Results obtained led to the establishment of a Square Root Function dependence as follows:

$${GP}_{\%}=71.85-1.911\sqrt{concentration\;of\;nano\;silver,\;\%\;v/v},$$

which had a statistical significance (*p* ≤ 0.01) with a coefficient of determination R^2^ = 51.988 and uncertainty K^2^ = 48.012. A strong negative correlation dependence (r = −0.721) in relation to the studied indicators (concentration of colloidal nano silver and laboratory seed germination) was also demonstrated.

Data from biometric measurements of root, hypocotyl and seedling length (cm) allowed the objective comparison and evaluation of the influence of the applied concentrations of colloidal nano silver on the initial development of the test plant (Table 3). The applied concentrations from 0.20 to 100.0% *v*/*v* exerted a statistically significant inhibitory effect on the initial development of *L. sativa*, both in terms of the root and shoot length, as well as on the seedling growth, when compared to the control (untreated) variant (*p* ≤ 0.001). An exception was found only for the lowest concentration of 0.10% *v*/*v*, where an indifferent, statistically unproven inhibitory effect was observed for all biometric measurements (root, hypocotyl and seedling).

Similar results were obtained in relation to the reduction of the studied parameters (R) and the percentage of inhibition (*I_%_*) for *L. sativa* growth, with the difference that the percentage of inhibition (*I_%_*) in the root length was from 0.3 to 2.4 times higher compared to that of hypocotyl length (Table 3).

**Figure 2:**
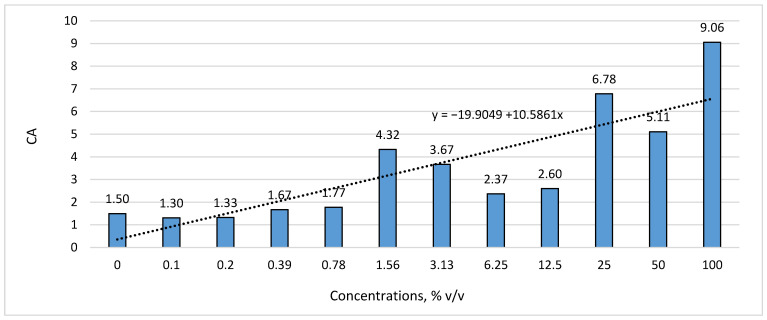
The effect of the nano silver on the coefficient of allometry (CA) of the test plant.

The biological impact of the applied concentrations of nano silver can be expressed as a change in the morphological indicators of the test plants by using the coefficient of allometry (*CA*). It can be seen from Figure 2 that as the concentration of colloidal nano silver in the experimental solutions increased, the coefficient of allometry (*CA*) increased from 0.87 to 6.05 times compared to the control variant with re-distilled water. The highest *CA* values (from 5.11 to 9.06, mean 6.98) were found at the highest concentrations of colloidal nano silver (from 25.0 to 100.0% *v*/*v*), followed by the concentrations between 1.56 and 12.50% *v*/*v*, where *CA* ranged from 2.37 to 4.32, with an average of 3.24. At the lower applied concentrations—from 0.10 to 0.78% *v*/*v*, *CA* kept relatively close values (from 1.3 to 1.77, average 1.52) compared to the control (untreated) variant (*CA* = 1.50), which shows a relatively equal isometric increase of the vegetative organs (root and hypocotyl) of the test plant *L. sativa*.

According to Niklas [45] and Niklas and Enquist [46], the coefficient of allometry (*CA*) can be used to express quantitative interrelationships that are genetically determined between the growth of roots and aboveground biomass, which retain approximately the same values for each plant species. Its change under the influence of chemical and/or allelopathic substances allows it to be used as a criterion for determining phytotoxic effects in the initial stages of the ontogenetic development of test plants when performing screening studies under laboratory conditions.

As a result of the one-factor regression analysis performed between the coefficients of allometry (*CA*) for the test plant (y) and the applied concentrations of colloidal nano silver (x), a linear and statistically significant (*p* ≤ 0.01) positive correlation dependence r = 0.865 with a coefficient of determination R^2^ = 74.855 and uncertainty K^2^ = 25.145 was found. This is evidence of a proportional relationship, i.e., as the concentration of colloidal nano silver in the solutions increased, the allometry coefficient increased, proving the inhibitory effect of the carrier on root growth in *L. sativa* (Figure 2).

**Figure 3:**
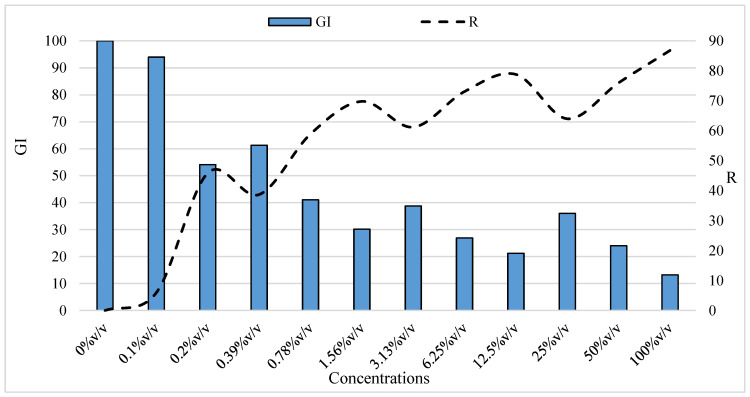
Integral effect of nano silver on the global development index (GI) of the test plant.

Analysis of the integral influence was the next step in the assessment of the biological effects of nano silver on the test plant, including the global development index (GI), calculated by average values of seed germination and sprout length (root + hypocotyl) (Figure 3). The significant differences in the cumulative value of GI and high reduction rate (R) of GI at the applied higher concentrations (from 0.20 to 100.0% *v*/*v*), as well as the insignificant reduction (R) of GI at the lowest concentration of colloidal nano silver (0.10% *v*/*v*), give us a reason to assume that colloidal nano silver at a concentration of 0.10% *v*/*v* can be successfully used when performing plant bioassays in laboratory conditions.

## 4. Discussion

Allelopathy has been widely studied from more than 50 years [47,48,49], and recently it was included in the category of sustainable agricultural practices, which are defined as practices that conserve resources and are organic, alternative, restorative, biodynamic and low cost [50,51]. Despite the attention paid to allelopathy by ecologists, biologists and herbologists, the complicated relationship of “competition–allelopathy” in the system “weed–crop plant” is not fully understood. Allelopathic relationships in agrophytocenoses are determined by a variety of factors occurring simultaneously and/or sequentially, having direct or indirect effects on plant species through the synthesis of various chemical substances (allelochemicals) released into the environment [48,52,53,54].

Many authors have revealed at contradictory results, confirming the inhibitory or stimulatory effects of various plants on the seed germination of other plant species [52,53,54,55]. The difficulties in such studies are found to be due to the lack of unifying methods for performing laboratory and field experiments, including those for growth media, extract preparation, microbial contamination, etc. [10,12,17,52,56,57]. Among these difficulties, probably the most serious are the challenges for the conservation of the activity and microbial prevention of extracts. The application of nano silver could address both of these problems, as silver is well known as a safe and effective bactericidal metal because it is non-toxic to animal cells and highly toxic to bacteria [18,45]. Silver nanoparticles are one of the most commonly used nano-materials in coating or embedding for medical purposes, as well as in clothing, food industry, paints, electronics and other fields [19,20,58]. Due to their properties, such as their high surface area, very small size and high dispersion, they can deeply penetrate into different living tissues, such as the blood–brain barrier and plant tissues [25,26]. In this way, if they do not exert an effect on test plants, they could accelerate allelopathic interactions and enhance the extent of allelopathic activity in laboratory screening studies.

One of the most common consequences reported into the literature is the inhibition of seed germination and suppression of seedling development. In the present laboratory experiments, we aimed to assess the effect of colloidal nano silver on these two processes, thus evaluating its possible application in allelopathic screening studies. The lowest concentration of nano silver (0.10% *v*/*v*) was found to stimulate germination, while the concentrations from 0.20 to 0.78% *v*/*v* had a weaker to indifferent inhibitory effect on it. When regarding the initial plant development, an indifferent, statistically unproven inhibitory effect was observed also in the lowest concentration of nano silver for all biometric measurements (root, hypocotyl and seedling). The established differences in the reduction index (R) and the percentage of inhibition (*I_%_*) at higher concentrations of nano silver can be explained by the increased content of silver nanoparticles in the test solutions, which probably induced oxidative stress and damaged cell membranes [58,59,60], thus resulting in a depressing effect on the seed germination and initial development of *L. sativa*. Similar results have been reported by other authors [32,61,62], according to which the increasing concentrations of silver nanoparticles significantly reduced the seed germination of *Brassica rapa* ssp. *rapa* L., *Lepidium sativum* L., *Trifolium repens* L. and *Pimenta dioica* Merr. when used as test plants.

The obtained experimental results in the present study are in agreement with those reported by Budhani et al. [30]. According to these authors, the use of nano solutions can cause a phytotoxic effect (depending on the concentration of silver nanoparticles in them), expressed as reducing the laboratory germination of seeds and/or suppressing the growth of plants in the initial stages of their development. The main reason for these effects is the accumulation of silver nanoparticles in the roots and/or leaves, which induces morphological changes in plant organisms and has an effect at the cellular and tissue levels, possibly altering metabolism and suppressing photosynthesis and/or transpiration in plants. However, Krishnaraj et al. [63] concluded that the migration of nanoparticles in plant organisms, as well as their cumulative accumulation in plant tissues, is a prerequisite for their use in laboratory screening studies. Our results correlate with the findings of Prazak et al. [64] that the biological impact of nano silver varies depending on the applied concentration of nanoparticles. These authors stated that the application of modern strategies, corresponding to the principles of precision agriculture and involving the controlled application of silver nanoparticles, could be effectively used for the pre-sowing treatment of the seeds of *Phaseolus vulgaris* L.

## 5. Conclusions

The experimental concentration of 0.10% *v*/*v* colloidal solution of nano silver exerted no significant effect on seed germination and plant growth (root, hypocotyl and total seedling length), as well as on the global development index (*GI* = 94.0%), of the test plant *Lactuca sativa* L. Therefore, it can be successfully applied as an allelochemical carrier in semi-solid agar (used as the growth medium of the test plant, *L. sativa*), suggesting better migration and/or contact of the allelochemicals with the test plant (due to the higher total surface area of the nanoparticles), and would help to establish allelopathic interference in the initial stages of plant development. Complex analyses revealed that this solution (the commercial product Silver–Amber©, diluted with redistilled water) can be successfully used for allelopathic screening studies under laboratory conditions at a concentration of 0.10% *v*/*v*. Further research, including validation in greenhouse conditions, is needed to establish the effect of colloidal nano silver in combined application with aqueous extracts or hydrolates of plants with allelopathic potential. Many variables are involved in such studies, i.e., the nanoparticles’ size, shape, concentration, ambient conditions, plant species, etc. Any of these factors could potentially contribute to varied outcomes, so these crucial parameters are needed to be carefully examined.

## Figures and Tables

**Table 1:** Description of the parameters used for the assessment of nano silver’s effect on the seed germination and initial development of *Lactuca sativa* L.

Parameter/Reference	Formula	Explanation
Percentage of germinated seeds, *GP_%_* Saxena et al. (1996) [39]	${GP}_{\%}=\left(\frac{NSG}{TNS}\right)\times 100$	*NSG*—number of germinated seeds *TNS*—total number of seeds used in all experimental variants and replicates
Length of root, hypocotyl and seedling, cm Golubinova et al. (2020) [40]	$SL=\sum_{i=1÷20}^{n}\frac{I}{n}$	*I*—number of individual measurements of plant organs for all experimental variants and replicates *n*—number of all measurements
Percentage of inhibition, *I_%_* Golubinova et al. (2020) [40]	$I_{\%}=100-\left(\frac{E_{2}}{E_{1}}\times 100\right)$	*E_1_*—response of plant seeds into the control *E_2_*—response of plant seeds from experimental variants
Reduction of studied parameters, *R* Thabet et al. (2018) [41]	$R=G_{c}-C_{i}$	Average values of biometric indicators of: *G_i_*—experimental variants *G_c_*—control (untreated) variant
Coefficient of allometry, *CA* Nasr and Mansour (2005) [42]	$CA=\frac{L_{s}}{L_{r}}$	*L_s_*—hypocotyl length, cm *L_r_*—root length, cm
Global development index, *GI* Gariglio et al. (2002) [43]	$GI=\left[\left(\frac{G}{G_{0}}\right)\times \left(\frac{L}{L_{0}}\right)\right]\times 100$	*G* and *G_0_*—germinated seeds in the experimental variants and the control (%); *L*—seedling length in the experimental variants; *L_0_*—seedling length in the control variant, taken as 100%

**Table 2:** Effect of the applied concentrations of colloidal nano silver on seed germination.

Concentration, % (*v*/*v*)	Percentage of Germinated Seeds, *GP_%_*	±Standard Error, SE	Reduction of Percentage Germinated Seeds, *R*	Percentage of Inhibition, *I_%_*
0.0 *	77.1 cd	2.4	0.0	0.0
0.10	83.2 е	3.75	−6.1	−7.91
0.20	71.6 bc	2.89	5.5	7.13
0.39	71.6 bc	2.04	5.5	7.13
0.78	69.1 c	2.30	8	10.38
1.56	60.0 a	3.48	17.1	22.18
3.13	63.4 ab	3.75	13.7	17.77
6.25	60.0 a	2.89	17.1	22.18
12.5	63.4 ab	4.04	13.7	17.77
25.0	63.4 ab	2.31	13.7	17.77
50.0	56.8 a	3.21	20.3	26.33
100.0	56.8 a	2.15	20.3	26.33

* Control (re-distilled water); a, b, c, d, e—LSD at statistical significance at *p* ≤ 0.001.

**Table 3:** Effect of different concentrations of nano silver on the initial development of *Lactuca sativa* L. (laboratory experiment).

Parameter	0.0 * % *v*/*v*	0.10% *v*/*v*	0.20% *v*/*v*	0.39% *v*/*v*	0.78% *v*/*v*	1.56% *v*/*v*	3.13% *v*/*v*	6.25% *v*/*v*	12.5% *v*/*v*	25.0% *v*/*v*	50.0% *v*/*v*	100.0% *v*/*v*
Root, cm	1.60 e	1.51 de	1.00 cd	0.99 cd	0.66 bc	0.31 ab	0.38 ab	0.38 abc	0.29 ab	0.22 ab	0.21 ab	0.07 a
±SE	0.13	0.18	0.20	0.20	0.18	0.22	0.20	0.25	0.21	0.20	0.20	0.22
*R*	0.00	0.09	0.6	0.61	0.94	1.29	1.23	1.22	1.31	1.38	1.39	1.53
*I_%_*	0.00	5.6	37.5	38.3	58.8	80.4	76.6	76.3	82.1	85.9	86.7	95.6
Hypocotyl, cm	2.39 d	1.97 cd	1.32 abc	1.65 bcd	1.17 abc	1.36 abc	1.38 abc	0.90 ab	0.74 ab	1.53 abc	1.09 ab	0.64 a
±SE	0.21	0.29	0.32	0.32	0.28	0.34	0.32	0.4	0.34	0.32	0.32	0.34
*R*	0.00	0.42	1.07	0.74	1.22	1.04	1.02	1.49	1.65	0.87	1.31	1.75
*I_%_*	0.00	17.7	44.7	31.1	51.1	43.3	42.6	62.4	69.0	36.3	54.6	73.1
Seedling, cm	3.99 e	3.48 de	2.33 bcd	2.64 cd	1.83 abc	1.67 abc	1.75 abc	1.28 abc	1.03 ab	1.75 abc	1.30 abc	0.71 a
±SE	0.33	0.44	0.49	0.49	0.44	0.52	0.50	0.63	0.53	0.50	0.49	0.52
*R*		0.51	1.67	1.36	2.16	2.32	2.24	2.71	2.97	2.24	2.69	3.28
*I_%_*	*I*_%_	12.9	41.8	34.0	54.2	58.2	56.2	68.0	74.2	56.2	67.5	82.1

* Control (re-distilled water); a, b, c, d, e—LSD at statistical significance at *p* ≤ 0.001; ±SE—standard error; *R*—reduction of percentage of the growth of root, hypocotyl and seedling; *I_%_*—percentage of inhibition.

## Data Availability

Dataset available on request from the authors.
